# Female sex worker’s participation in the community mobilization process: two distinct forms of participations and associated contextual factors

**DOI:** 10.1186/1471-2458-14-1323

**Published:** 2014-12-24

**Authors:** Karikalan Nagarajan, Seema Sahay, Mandar K Mainkar, Sucheta Deshpande, Sowmya Ramesh, Ramesh S Paranjape

**Affiliations:** National AIDS Research Institute- ICMR, 73, G Block, Pune, Bhosari, 411026 India; Population Council 1st Floor, 142 Golf Links, New Delhi, 110003 India

**Keywords:** Female Sex Workers, Participation, Community mobilization, Empowerment, HIV, Public spaces, Collective spaces, India

## Abstract

**Background:**

Community mobilization is a participatory intervention strategy used among Female Sex Workers (FSW's) to address HIV risks through behavior change and self empowerment. This study quantitatively measure and differentiate theoretically defined forms of FSW participation's and identify their contextual associated factors.

**Method:**

Data was derived from cross-sectional Integrated Bio Behavioral Assessment conducted among FSW’s in Andhra Pradesh (AP) (n = 3370), Maharashtra (MH) (n = 3133) and Tamil Nadu (TN) (n = 2140) of India during 2009–2010. Information’s about socio-demography, community mobilization and participation experiences were collected. Conceptual model for two contexts of mobilization entailing distinct FSW participations were defined as participation in “collective” and “public” spaces respectively. Bivariate and multiple regression analysis were used.

**Result:**

The level of participation in “collective” and “public” spaces was lowest in MH (43.9% & 11.7% respectively), higher in TN (82.2% & 22.5% respectively) and AP (64.7% & 33.1%). Bivariate and multivariate regression analysis highlighted the distinct nature of “participations” through their varied associations with FSW mobilization and background status.

In MH, street FSWs showed significantly lower collective participation (36.5%) than brothel FSWs (46.8%) and street FSWs showed higher public participation (16.2%) than brothel FSWs (9.7%). In AP both collective and public participation were significantly high among street FSWs (62.7% and 34.7% respectively) than brothel FSW’s (55.2% and 25.4% respectively).

Regression analysis showed FSWs with “community identity”, were more likely to participate in public spaces in TN and AP (AOR 2.4, 1.5-3.8 & AOR 4.9, CI 2.3-10.7) respectively. FSWs with “collective identity” were more likely to participate in collective spaces in TN, MH and AP (AOR 27.2 CI 13.7-53.9; AOR 7.3, CI 3.8-14.3; AOR 5.7 CI 3–10.9 respectively). FSWs exhibiting “collective agency” were more likely to participate in public spaces in TN, MH and AP (AOR 2.3 CI 1–3.4; AOR 4.5- CI 2.6-7.8; AOR 2.2 CI 1.5-3.1) respectively.

**Conclusion:**

Findings reveal FSWs participation as a dynamic process inherently evolving along with the community mobilization process in match with its contexts. Participation in “Collective” and Public spaces” is indicators, symbolizing FSWs passage from the disease prevention objectives towards empowerment, which would help better understand and evaluate community mobilization interventions.

## Background

Community mobilization is a widely used intervention strategy to address crucial public health issues such as HIV among high risk groups of Female Sex Workers (FSWs) worldwide [1, 2]. Community mobilization is conceptualized as a ‘continuous process’ with sequential stages of development, which function under two distinct contexts [3, 4]. The first context refers to initial ‘collectivization’ of FSWs which aims to bring in individual behavior change among them for HIV prevention and risk aversion [4, 5]. The second context refers to ‘community engagement’ which aims at societal empowerment of FSWs [3, 6]. These two types of contexts entail two types of participations of FSWs respectively: 1) In ‘Collectivization’ where the ‘participation’ of FSWs is within their own sex work settings called as ‘collective spaces’, 2) In ‘Community engagement’ where the ‘participation’ of FSWs goes beyond their sex work setting/collective space to broader ‘public or social spaces’.

The distinct and transformative nature of ‘community participations’ has been theorized and assessed with regard to health and development programmes [7]. But still there is a gap in assessing different forms, strategies, and objectives of participations in the context of community based HIV prevention initiatives in India [8]. Studies in India have widely assessed FSWs’ ‘exposure’ to intervention activities like ‘peer education’ in the community mobilization process [9]. Such assessments done from the passive perspective of ‘programme exposures’ tend to ignore the dynamic, distinct and active ‘participatory’ process of FSWs in their real empowerment process. In India, till date only one qualitative case study about Sonagachi community mobilization project had defined and identified four domains of participations among FSWs [10]. The participatory domains identified in Sonagachi project were mostly the utilitarian context of community mobilization that occurred within the collective spaces of FSW’s.

According to a recent report based on large scale community based intervention program called AVAHAN in India, in the year 2007 the number of FSWs reported to be 115,000, 72,000 and 84,000 FSWs in the three high prevalence states of Andhra Pradesh (AP), Maharashtra (MH), and Tamilnadu (TN) respectively [11]. According to this report, in the MH state, total FSWs were covered by the community mobilization interventions in which 74% were implemented by AVAHAN and rest were covered under government initiatives. In AP and TN 61% and 36% of FSWs were covered by AVAHAN respectively while 29% and 38% were covered by government initiatives. Of all, 11% FSWs in AP and 26% FSWs in TN were not covered by any community mobilization program till 2008.

Although, community mobilization is widely used intervention among FSWs; the assessments about their actual form/s of ‘participation/s’ has been limited. The ‘participations of the beneficiary community’ can be strong evaluation indicators of empowerment programs. Community mobilisation intervention under AVAHAN program was implemented in MH, AP and TN with the goal of HIV prevention and empowerment of sex worker. This approach of community mobilization could comprise of various types of ‘FSW participations’. We present the first study on quantitative assessment of the two distinct forms of participations in the prevention and empowerment context of FSWs in the states of AP, TN and MH in India: 1) participation in collective spaces and 2) participation in public spaces. Study also identifies the contextual factors associated with these two types of participations.

## Methods

### Study design and sampling

Data was derived from the cross-sectional Integrated Bio Behavioral Assessment (IBBA) survey which was conducted among FSWs in eight districts of Andhra Pradesh (AP), six districts of Maharashtra (MH) and five districts of Tamil Nadu (TN) state in India between 2009 and 2010. This survey was conducted in FSW intervention target districts in three states as part of the AVAHAN program. The survey objective was to exclusively measure the major outcomes and impacts of community led HIV interventions under AVAHAN. While the AVAHAN community led intervention was initiated in the year 2003, it was systematically scaled up to cover 65% of the target population in all target states by late 2005 and further extended to reach 70-90% by the year 2007. Thus the IBBA data used in this study was collected almost one year after the maximum implementation of the community mobilization intervention among FSW populations [11, 12]. A representative sample of 400 FSWs was selected per district through two-stage cluster sampling using probability proportional to size method at first stage and simple random sampling at the second stage. The inclusion criteria for survey participants was defined as ‘any female 18 years or older, either brothel based or non brothel based, who sold sex in exchange for cash at least once in the last one month’. Fixed-location and time-location clusters were used as the primary sampling units in brothel and non brothel sites in all districts respectively. A total of 8643 respondents were covered in the survey with 3370 in AP, 3133 in MH and 2140 in TN [12]. The survey was approved by the ethics committees of participating institutes of Indian Council of Medical Research (ICMR) and Family Health Internationsla (FHI’s) Protection of Human Subjects Committee. Written informed consent obtained from the respondents before the administration of structured questionnaire. The socio demographic characteristics of FSWs including age, literacy status, state of residence, occupation, marital status, and duration of sex work, typology of sex and debt status were collected. Data was also collected on two mobilization contexts viz. ‘collectivization’ and ‘community engagement”’ and forms of participations. Contextual variables related to the violence and vulnerability experienced by FSWs was also collected.

### Framework of community mobilization and participation

**Figure 1:**
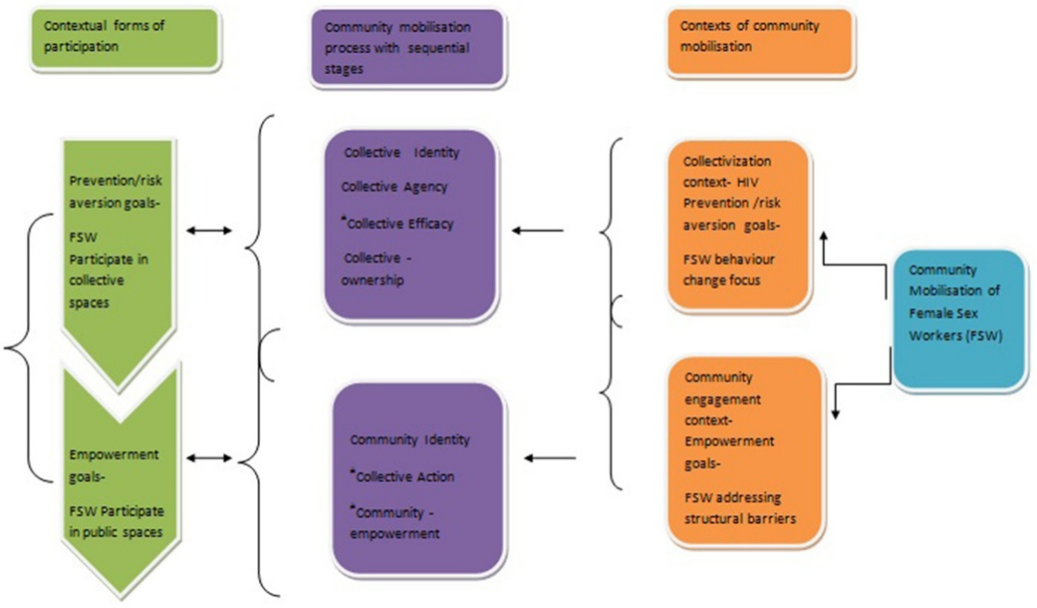
**Framework of community mobilization featuring two forms of FSW participation.** ^a^indicators not included in analysis.

Community mobilization process of FSWs has two contexts of collectivization and community engagement with focus on 1) HIV prevention and risk aversion among FSWs and 2) Empowerment of FSWs (Figure 1). In both the contexts of community mobilization process, FSWs pass through distinct ‘stages’ to meet their contextual goals. The stages may overlap or may not be sequential during the entire course of mobilization process. The stages for two distinct contexts are delineated as follows:

Stages of collectivization context are: 1. Collective identity, 2. Collective agency, 3. Collective efficacy, and 4. Collective ownership. Community mobilization in HIV prevention context among sex workers starts with collectivization processes where ‘collective identity’ of sex workers is formed through relationship ties in their safe ‘collective spaces’. This further evolves into ‘collective agency and efficacy’ which reflects FSWs’ confidence in their power of the community to work together for positive changes and their action to claim their rights [13]. Following this, FSWs gain ownership of the collectives, where they have power, influence and accountability over activities which they undertake [3, 14]. The process of participation by sex workers in these stages of collectivization are within the collective spaces and the activities could be peer education classes, condom distribution, trainings and counseling [6, 10]. This ‘participation in collective spaces’ is participation with an instrumental value, where participation is a ‘utilitarian tool’ with health promotion, safety and prevention as focus [7, 15].

Stages of “community engagement” context are: 1. Community identity, 2. Collective action, and 3. Community empowerment. In this context the sex workers engage in a broader functional space, by attaining ‘community identity’ by creating relationship ties with wider community based organizations and they build organizational resilience towards structural barriers through their organized collective actions [4]. Further this stage leads to the actual ‘community empowerment’ of FSWs in the society where they might enjoy their due rights and entitlements. In this context of community mobilization, FSWs participate in wider ‘public spaces’ which would be distinct from their previous forms of participation within the collective spaces. This ‘public participation’ features as a crucial step for attaining ‘empowerment’. This participation in public spaces could be theoretically explained as ‘participation with an intrinsic value’, as a tool of empowerment [6, 7].

Variables used to indicate stages of community mobilization within the two contexts: Based on the proposed framework (Figure 1), the concepts of community mobilization and participations, following variables were defined for this study.

‘Collective identity’ was defined as the shared sense of oneness developed among people with shared identity within the collective spaces. Membership of self help group was used to define ‘collective identity’. Negotiation with someone in power to protect fellow sex worker was used to define ‘collective agency’. Participation in HIV education classes or counseling or trainings organized by an NGO in the past six months was used to define ‘participation in collective spaces’. The fourth variable was’collective ownership’ which brings empowerment among FSWs. This was defined as being peer or unpaid volunteer working in HIV prevention services of an NGO. Being a member of a Community Based Organisation (CBO) has been used to define the ‘community identity’ which reflects shared sense of oneness developed among people with shared identity outside collective spaces in the wider community spaces. ‘Participation in public spaces’ was defined as an active role played by a sex worker openly expanding her consciousness and activities, without hiding her identity in public spaces, thereby influencing decision-making which impacts her life. It was measured by asking whether in the past six months the sex worker had participated in a public event (like gatherings, rallies) where she could be identified as sex worker.

In addition to the community mobilization status of FSWs, the vulnerability and violence faced by FSWs in their sex work settings is considered as a potential ‘push’ factor towards their public participation in this study. FSWs, which experience more vulnerability, would be naturally tending towards a ‘public participation spaces’ where they could find a wider support and unity.

Thus two measures of potential vulnerability and violence faced by FSWs were included in this study. As per the definition of by United Nations [16] “experience of violence was measured if the FSWs were beaten, hurt, hit, slapped, pushed, kicked, punched, choked, burnt without weapon and physically forced to have sexual intercourse. WHO defines vulnerability as ‘emotional or psychological violence as one which includes, but is not limited to, being insulted or made to feel bad about oneself; being humiliated or belittled in front of other people; being threatened with loss of custody of one’s children; being confined or isolated from family or friends….controlling behaviour; and the destruction of possessions’ [17]. Using the component of confinement or isolation from this definition, FSW’s arrest experience by police has been considered equal to violence/vulnerability in this study.

### Statistical methods

All the analysis of this study was done separately for the three states of Tamil Nadu (TN), Maharashtra (MH) and Andhra Pradesh (AP) considering the regional socio-cultural differences, and other differences in viz. demography, geography, and sex work settings of FSWs. It was also intended to check the consistency of emerging findings through comparison between the states.

Descriptive was used assess the level of participations and community mobilization among FSWs across states. Bivariate analyses were used to assess the associations between public and collective participation of FSWs with their socio-demographic characteristics. Multiple logistic regression analysis was used to identify factors associated with FSWs’ participation in public places and collective spaces. A dichotomous dependant variable was created and coded as ‘1’ for participation and coded as ‘0’ for non participation of FSWs. The variables defined to represent the successive stages of community mobilization in this study were the independent variables. In addition, the variables of violence and vulnerability which were logically considered to influence public participation of FSWs were used in both models to comparatively identify their influence on both types of participations. Thus the measures of collective identity, collective agency, collective ownership, community identity, police vulnerability and experience of violence were included as independent variables. Further, both types of participations were used as independent variables to identify their relations with each other. All the independent factors were adjusted for sample characteristics like age, literacy, typology of sex, debt status and marital status. The multivariate analysis was performed separately for three states and all analysis were performed after adjusting for sampling differences by applying appropriate sampling weights. Adjusted odds ratio was calculated at significance level less than 0.05. STATA/SE version 12.0 was used for performing all analysis.

## Results

**Figure 2:**
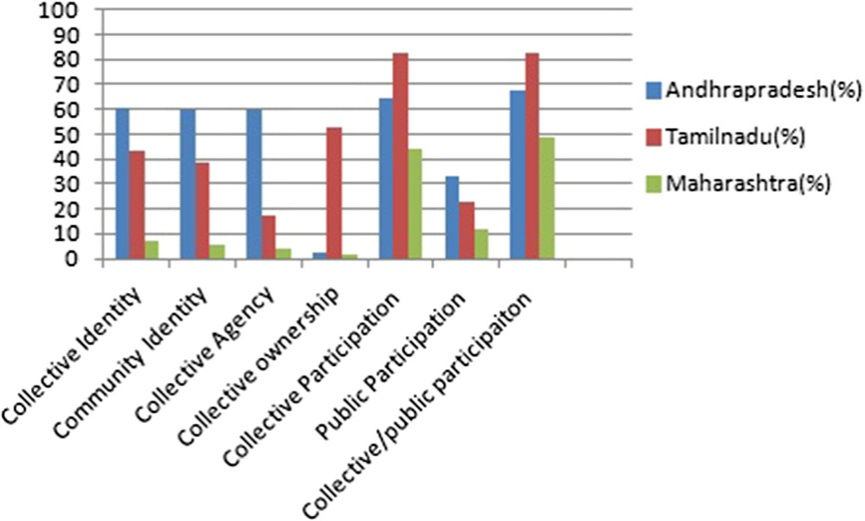
**Community mobilization status and participations of FSWs in the states of Andhra Pradesh, Tamil Nadu and Maharashtra in India.**

Figure 2 indicates the various stages of community mobilization and participation exhibited by FSWs in all three states.

### Community mobilization status of FSWs

It shows that collective and community identity were higher in AP (60.3% & 59.8% respectively), moderate in TN(43.6% & 38.2% respectively) and lowest in MH(6.9% & 5.7% respectively). Exhibition of collective agency was high in AP (59.4%), moderate in TN (17.5%) and least in MH (4.3%). Collective ownership was high in TN (52.9%), very low in AP and MH (2.4% and 2% respectively).

### Participation status of FSWs

Participation in collective spaces was higher in TN and AP (82.2% & 64.7% respectively), and it was reported nearly by half of the FSWs in MH (43.9%). In case of participation in public spaces it was generally lower in all the states, with MH the least (11.7%), and TN and AP with moderate level (22.5% &33.1% respectively). FSWs who participated either in public or collective spaces were 82.7% in TN, 68% in AP and 48.9% in MH.

Bivariate analysis in Table 1 shows the FSWs background characteristic associated with collective participations.

**Table 1:** **Participation in collective spaces by background characteristics among FSW in Andhra Pradesh (AP), Tamil Nadu (TN) and Maharashtra (MH), India**

FSW characteristics in AP		Participation in collective spaces in AP	FSW characteristics in TN		Participation in collective spaces in TN	FSW characteristics in MH		Participation in collective spaces in MH
(N = 3370)		Yes(N = 2237)	(N = 2140		Yes (N = 1633)	(N = 3133)		Yes (N = 1465)
(n)		n(%)	(n)		n(%)	(n)		n(%)
**Age (yrs)**	18-25(947)	539(55.8)*	**Age (yrs)**	18-25(318)	214(70.9)*	**Age (yrs)**	18-25(1034)	442(37.7)*
	26-30(978)	691(68.7)		26-30(468)	350(81.8)		26-30(839)	371(44.7)
	31-35(703)	498(66.9)		31-35(466)	366(85.3)		31-35(546)	271(47.3)
	>35(742)	509(71.1)		>35(888)	703(84.4)		>35(714)	381(50.4)
**Duration of sex work(yrs)**	0-2(1016)	549(51.5)*	**Duration of sex work(yrs)**	0-2(593)	397(73.3)*	**Duration of sex work(yrs)**	0-2(1007)	409(38.3)*
	3-5(1218)	830(65.5)		3-5(839)	665(83.7)		3-5(856)	402(45.7)
	6-45(1136)	858(76.5)		6-45(708)	571(87.1)		6-45(1270)	654(46.7)
**Typology**	Street(1995)	1256(62.7)*	**Typology**	Street(2045)	1557(81.8)	**Typology**	Street(1219)	493(36.5)*
	Brothel(248)	168(55.2)		Brothel(29)	26(94.1)		Brothel(1657)	824(46.8)
	Others(1127)	813(71.4)		Others(66)	50(79.4)		Others(257)	148(53.3)
**Married**	Yes(2274)	1562(66.0)*	**Married**	Yes (1677)	1312(84.0)*	**Married**	Yes (1706)	782(43.0)
	No(1096)	675(61.9)		No(463)	321 (75.4)		No(1427)	683(45.1)
**Exclusive sex work occupation**	Yes(1709)	1050(62.8)*	**Exclusive sex work occupation**	Yes(476)	326(74.3)*	**Exclusive sex work occupation**	Yes (2691)	1246(44.2)
	No(1661)	1187 (66.8)		No(1664)	1307(84.3)		No(442)	219(41.5)
**Literates**	Yes(1437)	947(64.2)	**Literates**	Yes(1173)	912(83.6)	**Literates**	Yes (854)	422(50.8)
	No (1933)	1290(65.1)		No(967)	721(80.1)		No(2279)	1043(41.6)
**In Debt**	Yes(2631)	1804(65.9)*	**Debt**	Yes (1604)	1270(83.0)*	**Debt**	Yes (1345)	603(41.2)
	No (739)	433(61.1)		No(536)	363(79.7)		No(1788)	862(46.4)
**Collective Identity**	Yes(2026)	1839(89.3)*	**Collective Identity**	Yes (811)	793(98.9)*	**Collective Identity**	Yes (243)	215(87.8)*
	No (1344)	398(27.2)		No (1329)	840(69.2)		No(2890)	1250
**Community Identity**	Yes(1994)	1810(89.4)*	**Community Identity**	Yes(941)	780(87.8)*	**Community Identity**	Yes (178)	142(79.6)*
	No (1376)	427(28.0)		No(1199)	853(78.7)		No(2955)	1323(41.7)
**Collective agency**	Yes(1918)	1526(76.6)*	**Collective agency**	Yes (293)	261(94.9)*	**Collective agency**	Yes (153)	86(58.4)*
	No (1452)	711(47.3)		No (1847)	1372(79.5)		No(2980)	1379(43.3)
**Collective ownership**	Yes(97)	93(98.0)	**Collective ownership**	Yes (942)	888(96.1)*	**Collective ownership**	Yes (99)	94(89.8)*
	No (3273)	2144(63.9)		No (1198)	745(66.5)		No(3034)	1371(43.0)
**Police Vulnerability**	Yes(1030)	832(80.2)*	**Police Vulnerability**	Yes (143)	114(85.1)	**Police Vulnerability**	Yes (1284)	609(49.4)
	No (2340)	1405(57.4)		No(1997)	1519(82.0)		No(1849)	856(39.2)
**Experience of Violence**	Yes(918)	709(78.7)*	**Experience of Violence**	Yes (294)	216(77.5)	**Experience of Violence**	Yes (501)	229(39.3)
	No (2452)	1528 (58.6)		No (1846)	1417(82.9)		No(2630)	1235(44.8)

*p < .05 Chi Square test.

### FSW characteristics associated with collective participation

FSWs with higher age and duration of sex had a significantly high proportion of collective participation in AP and TN (60% to 85% range) and in MH it was comparatively low (45 to 50% range). Significantly high proportion of FSWs who participated in collective spaces were married in AP (66.0%) and in TN (84.0%); were involved in exclusive sex work occupation in AP (62.8%) and in TN (74.3%); and were in debt status in AP(65.9) and in TN(83.0%). In MH, street based FSWs showed significantly lower collective participation (36.5%) than brothel based FSWs (46.8%) Alternatively in AP collective participation among street based FSWs was significantly higher (62.7%) than street based FSW’s (55.2%).

### FSWs community mobilization and vulnerability status associated with collective participation

FSWs who shown collective identity, collective ownership, collective agency and community identity were having significantly high proportion of collective participation in all states (>75%), except in MH where FSWs with collective agency were having relatively lesser collective participation (58.4%). FSW who experienced police vulnerability and violence were significantly having higher collective participation in AP state (>79%).

Bivariate analysis in Table 2 shows FSWs characteristics associated with public participation.

**Table 2:** **Participation in public spaces by background characteristics among FSW in Andhra Pradesh (AP), Tamil Nadu(TN) and Maharashtra(MH), India**

FSW characteristics in AP		Participation in public spaces in AP	FSW characteristics in TN		Participation in public spaces in TN	FSW characteristics in MH		Participation in public spaces in MH
(N = 3370)		Yes (N = 1218)	(N = 2140)		Yes (N = 472)	(N = 3133)		Yes(N = 429)
(n)		n (%)	(n)		n (%)	(n)		n (%)
**Age (yrs)**	18-25(947)	237(21.7)*	**Age (yrs)**	18-25(318)	43(14.1)*	**Age (yrs)**	18-25(1034)	108(9.7)*
	26-30(978)	362(34.9)		26-30(468)	99(25.3)		26-30(839)	113(13.0)
	31-35(703)	293(40.6)		31-35(466)	112(20.0)		31-35(546)	86(11.2)
	>35(742)	326(40.6)		>35(888)	218(25.3)		>35(714)	122(13.7)
**Duration of sex work(yrs)**	0-2(1016)	222(20.0)*	**Duration of sex work(yrs)**	0-2(593)	62(10.1)*	**Duration of sex work(yrs)**	0-2(1007)	106(8.2)*
	3-5(1218)	454(35.8)		3-5(839)	187(23.2)		3-5(856)	131(15.1)
	6-54(1136)	542(42.5)		6-45(708)	223(31.0)		6-45(1270)	192(12.1)
**Typology**	Street(1195)	757(34.7)*	**Typology**	Street(2045)	456(22.9)	**Typology**	Street(1219)	244(16.2)*
	Brothel(248)	79(25.4)		Brothel(29)	8(20.9)		Brothel(1657)	154(9.7)
	Others(1127)	382(31.1)		Others(66)	8(6.2)		Others(257)	31(13.1)
**Married**	Yes (2274)	837(33.6)	**Married**	Yes (1677)	372(21.6)	**Married**	Yes (1706)	250(12.0)
	No(1096)	381(32.1)		No(463)	100(26.1)		No(1427)	179(11.4)
**Exclusive sex work occupation**	Yes (1709)	509(27.4)*	**Exclusive sex work occupation**	Yes(476)	116(26.0)	**Exclusive sex work occupation**	Yes (2691)	327(11.2)*
	No(1661)	709(39.1)		No(1664)	356 (21.6)		No(442)	102(17.7)
**Literates**	Yes (1437)	514(30.9)	**Literates**	Yes(1173)	287(25.8)*	**Literates**	Yes (854)	138(16.3)*
	No(1933)	704(34.8)		No(967)	185(17.9)		No(2279)	291(10.2)
**Debt**	Yes ((2631)	1050(37.7)*	**Debt**	Yes(1604)	346(18.2)	**Debt**	Yes (1345)	194(12.4)
	No (739)	168(18.6)		No(536)	126(36.0)		No(1788)	235(11.2)
**Collective Identity**	Yes (2026)	1064(48.7)*	**Collective Identity**	Yes(811)	322(33.3)*	**Collective Identity**	Yes (243)	95(27.0)*
	No (1344)	154(9.4)		No(1329)	150(14.2)		No(2890)	334(10.6)
**Community Identity**	Yes(1994)	1056(49.3)*	**Community Identity**	Yes(941)	332(33.5)*	**Community Identity**	Yes (178)	74(26.8)
	No (1376)	162(9.0)		No(1199)	140(15.7)		No(2955)	355(10.8)
**Collective agency**	Yes (1918)	983(45.1)*	**Collective agency**	Yes(293)	127(46.6)*	**Collective agency**	Yes (153)	71(42.0)*
	No (1452)	235(15.5)		No(1847)	345(17.4)		No(2980)	358(10.4)
**Collective ownership**	Yes (97)	69(71.4)*	**Collective ownership**	Yes(942)	285(32.2)*	**Collective ownership**	Yes (99)	53(40.3)*
	No (3273)	1149(32.1)		No(1198)	187(11.7)		No (3034)	376(11.2)
**Police Vulnerability**	Yes (1030)	682(58.0)*	**Police Vulnerability**	Yes(143)	56(49.9)*	**Police Vulnerability**	Yes (1284)	242 (18.3)*
	No(2340)	536(21.3)		No(1997)	416(21.0)		No(1849)	187(6.0)
**Experience of Violence**	Yes (918)	493(45.8)*	**Experience of Violence**	Yes(294)	77(24.3)	**Experience of Violence**	Yes (501)	105(17.0)*
	No (2452)	725(27.5)		No(1846)	395(22.3)		No(2630)	324(10.8)

*p < .05 Chi Square test.

FSWs with higher age and duration of sex had a significantly moderate proportion of public participation in AP and TN (25% to 45% range) and very lesser proportion public participation in MH (10%-15% range). In MH, street based FSWs showed significantly higher public participation (16.2%) than brothel based FSWs (9.7%). In AP street based FSW had higher public participation (34.7%) than brothel based FSWs (25.4%). FSWs who involved in exclusive sex work occupation had significantly lesser public participation in AP and (27.4%) in MH (11.2%). FSWs who were literates were showing lower public participation in TN and MH (<25%).

### FSWs community mobilization and vulnerability status associated with public participation

FSWs that showed collective identity, collective ownership, collective agency and community identity were having moderate proportion of public participation in all states (27% to 50% range). FSW who experienced police vulnerability and violence were significantly having higher public participation in AP and TN (45%-59% range), while in MH they had significantly very low public participation (<20%).

Multivariate regression in Table 3 shows the likelihood odds of various factors associated with the “collective participation” of FSWs.

**Table 3:** **Multivariate analysis of ‘participation in collective spaces’ by FSW status of community mobilization and vulnerability in Andhra Pradesh Tamil Nadu and Maharashtra**

	Andhra Pradesh				Tamil Nadu				Maharashtra		
Characteristics(n)	Collective participation Yes (N = 2237)	OR (95% CI)	AOR (95% CI)	Characteristics	Collective participation Yes (N = 1633)	OR (95% CI)	AOR (95% CI)	Characteristics	Collective participation Yes (N = 1465)	OR (95% CI)	AOR (95% CI)
	n(%)				n(%)				n (%)		
**Collective identity**				**Collective identity**				**Collective identity**			
Yes(2026)	1839(89.3)	22.3(14.8-33.7)*	5.7(3–10.9)*	Yes(811)	793(98.9)	39.8(21.0-75.7)*	27.2(13.7-53.9)*	Yes(243)	215(87.8)	10.4(5.9-18.4)*	7.3(3.8-14.3)*
No(1344)	398(27.2)	Ref	Ref	No(1329)	840(69.2)	Ref	Ref	No(2890)	1250(40.7)	Ref	Ref
**Community identity**				**Community identity**				**Community identity**			
Yes(1994)	1810(89.4)	21.6(14.2-32.6)*	2.5(1.3-4.9)*	Yes(941)	780(87.8)	1.9(1.3-2.8)*	1.2(0.8-1.9)	Yes(178)	142(79.6)	5.4(2.9-9.9)*	1(0.6-4)
No(1376)	427(28.0)	Ref	Ref	No(1199)	853(78.7)	Ref	Ref	No(2955)	1323(41.7)	Ref	Ref
**Collective agency**				**Collective agency**				**Collective agency**			
Yes(1918)	1526(76.6)	3.6(2.5-5.1)*	1.2(0.8-1.8)	Yes(293)	261(94.9)	4.8(2.5-9.0)*	1.7(0.8-3.8)	Yes(153)	86(58.4)	1.8(1.1-2.9)*	0.8(0.4-1.4)
No(1452)	711(47.3)	Ref	Ref	No(1847)	1372(79.5)	Ref	Ref	No(2980)	1379(43.3)	Ref	Ref
**Collective ownership**				**Collective ownership**				**Collective ownership**			
Yes(97)	93(98.0)	28.9(10–83.0)*	8(2.2-28.3)*	Yes(942)	888(96.1)	12.6(8.2-19.3)*	9.6(6.1-15)*	Yes(99)	94(89.8)	11.7(3.9-35.3)*	4.1(1–17)*
No(3273)	2144(63.9)	Ref	Ref	No(1198)	745(66.5)	Ref	Ref	No(3034)	1371(43.0)	Ref	Ref
**Public space participation**				**Public Space participation**				**Public Space participation**			
Yes(1218)	1094(90)	8.2(5.4-12.3)*	2.8(1.6-4.8)*	Yes(472)	450(97.7)	12.3(6.4-23.5)*	4.3(1.9-9.4)*	Yes(429)	278(58.2)	1.9(1.2-3.0)*	1.4(0.8-2.3)
No(2152)	1143(52.2)	Ref	Ref	No(1668)	1183(77.7)	Ref	Ref	No(2704)	1187(42.0)	Ref	Ref
**Police vulnerability**				**Police vulnerability**				**Police vulnerability**			
Yes(1030)	832(80.2)	3.0(2.1-4.2)*	1.1(0.7-1.8)	Yes(143)	114(85.1)	1.2(0.6-2.3)	O.4(0.1-1.0)	Yes(1284)	609(49.4)	1.5(1.1-1.9)*	1.6(1.2-2.1)*
No(2340)	1405(57.4)	Ref	Ref	No(1997)	1519(82.0)	Ref	Ref	No(1849)	856(39.2)	Ref	Ref
**Experience of violence**				**Experience of violence**				**Experience of violence**			
Yes(918)	709(78.7)	2.6(1.8-3.7)*	1.4(0.9-2.2)	Yes(294)	216(77.5)	0.7(0.3-1.3)	1(05–1.8)	Yes(501)	229(39.3)	0.7(0.5-1.1)	0.7(0.4-1)
No(2452)	1528(58.6))	Ref	Ref	No(1846)	1417(82.9)	Ref	Ref	No(2630)	1235(44.8)	Ref	Ref

The dependant variable is created by coding 1 for participation in collective spaces and coded as 0 of not participated. *p < .05 CI- Confidence Interval; OR- Crude Odds Ratio; AOR-Adjusted Odds Ratio Adjusted for Age, Duration of Sex work, typology of sex, Occupation, Literacy and Debt status.

FSWs showing “collective identity” had high likelihood to participate in collective spaces in all the three states of AP, TN and MH consistently (AOR 5.7 CI 3–10.9: AOR 27.2 CI 13.7-53.9; AOR 7.3 3.8-14.3 respectively). Alternatively, FSWs that were having “community identity” had no association with their collective participation in the state of TN and MH, except in AP where an increased odd (AOR 2.5 CI 1.3-4.9) for collective participation was noted.

While “collective agency” of FSWs were not having any association with collective participation in any of the three states, FSWs who reported having “collective ownership” roles were found to be 4 to 9 times more likely to participate in collective spaces in all states of AP, TN and MH, (AOR 8 CI 2.2-28.3; AOR 9.6 CI 6.1-15; AOR 4.1 CI 1–17; respectively).

In TN and AP, those FSWs who were participating in public spaces, were more likely to participate in collective spaces (AOR 4.3 CI 1.9-9.4; AOR 2.8 CI 1.6-4.8 respectively). FSWs who had experienced “police vulnerability” showed no association with collective participation except in the state of MH, where increased odds for collective participation was observed (AOR 1.6 CI 1.2-2.1).

Multivariate regression in Table 4 shows the likelihood odds of various factors associated with the “public participation” of FSWs.

**Table 4:** **Multivariate analysis of ‘participation in public spaces’ by FSW status of community mobilization and vulnerability in Andhra Pradesh, Tamil Nadu and Maharashtra**

		Andhra Pradesh				Tamil Nadu				Maharashtra	
Characteristics(n)	Public participation Yes (N = 1218)	OR (95% CI)	AOR (95% CI)	Characteristics	Public participation Yes (N = 472)	OR (95% CI)	AOR (95% CI)	Characteristics	Public participation Yes (N = 429)	OR (95% CI)	AOR (95% CI)
	n(%)				n(%)				n(%)		
**Collective Identity**				**Collective identity**				**Collective identity**			
Yes(2026)	1064(48.7)	9.1(6.5-12.7)*	0.8(0.3-17)	Yes(811)	322(33.3)	3.0(1.8-4.8)*	1.4(0.9-2.4))	Yes(243)	95(27.0)	3.1(1.8-5.1)*	1.2(0.7-2.1)
No(1344)	154(9.4)	Ref	Ref	No(1329)	150(14.2)	Ref	Ref	No(2890)	334(10.6)	Ref	Ref
**Community Identity**				**Community identity**				**Community identity**			
Yes(1994)	1056(49.3)	9.8(7.0-13.6)*	4.9(2.3-10.7)*	Yes(941)	332(33.5)	2.6(1.5-4.5)*	2.4(1.5-3.8)*	Yes(178)	74(26.8)	3.0(1.6-5.4)	1(0.5-1.9)
No(1376)	162(9.0)	Ref	Ref	No(1199)	140(15.7)	Ref	Ref	No(2955)	355(10.8)	Ref	Ref
**Collective Agency**				**Collective agency**				**Collective agency**			
Yes(1918)	983(45.1)	4.4(3.2-6.1)*	2.2(1.5-3.1)*	Yes(293)	127(46.6)	4.1(2.1-7.8)*	2.3(1.3-4)*	Yes(153)	71(42.0)	6.2(3.6-10.7)	4.5(2.6-7.8)*
No(1452)	235(15.5)	Ref	Ref	No(1847)	345(17.4)	Ref	Ref	No(2980)	358(10.4)	Ref	Ref
**Collective Ownership**				**Collective ownership**				**Collective ownership**			
Yes(97)	69(71.4)	5.2(2.8-9.9)*	2.2(1–4.7)*	Yes(942)	285(32.2)	3.5(2.3-5.4)*	2.3(1.5-3.5)*	Yes (99)	53(40.3)	5.3(2.9-9.7)*	2.4(1.1-5.3)*
No(3273)	1149(32.1)	Ref	Ref	No(1198)	187(11.7)	Ref	Ref	No(3034)	376(11.2)	Ref	Ref
**Collective space participation**				**Collective space participation**				**Collective space participation**			
Yes(2237)	1094(46.0)	8.2(5.4-12.3)	2.9(1.7-5.0)*	Yes(1633)	450(26.8)	12.3(6.4-23.5)*	5.1(2.6-10.3)*	Yes(1465)	278(15.6)	1.9(1.2-3.0)*	1.4(0.9-2.4)
No(1133)	124(9.4)	Ref	Ref	No(507)	22(2.8)	Ref	Ref	No(1668)	151(8.7)	Ref	Ref
**Police Vulnerability**				**Police vulnerability**				**Police vulnerability**			
Yes(1030)	682(58.0)	5.0(3.5-7.3)	3.6(2.6-5.1)*	Yes(143)	56(49.9)	3.7(2.0-6.6)*	2.4(0.3-2.3)*	Yes(1284)	242(18.3)	3.4(2.3-5.1)*	3.3(2.1-5)*
No(2340)	536(21.3)	Ref	Ref	No(1997)	416(21.0)	Ref	Ref	No(1849)	187(6.0)	Ref	
**Experience of Violence**				**Experience of violence**				**Experience of violence**			
Yes(918)	493(45.8)	2.2(1.5-3.1)	0.9(0.6-1.3)	Yes(294)	77(24.3)	1.1(0.6-1.8)	0.8(0.4-1.6))	Yes(501)	105(17.0)	1.6(1.0-2.6)*	1.3(0.8-2.3)
No(2452)	725(27.5)	Ref	Ref	No(1846)	395(22.3)	Ref	Ref	No(2630)	324(10.8)	Ref	Ref

The dependant variable is created by coding 1 for participation in Public spaces and coded as 0 of not participated. *p < .05 CI- Confidence Interval; OR- Crude Odds Ratio; AOR-Adjusted Odds Ratio Adjusted for Age, Duration of Sex work, typology of sex, Occupation, Literacy and Debt status.

FSWs that were having “collective identity” had no association with their public participation in any of the states. Alternatively, FSWs who had “community identity” were 2 to 5 times more likely to participate in public spaces in the state of AP and TN (AOR 4.9 CI 2.3-10.7; AOR 2.4 CI 1.5-3.8 respectively).

FSWs, who demonstrated “collective agency”, were 2 to 4 times more likely to participate in public spaces consistently in all three sates of AP, TN and MH, (AOR 2.2 CI 1.5-3.1; AOR 2.3 CI 1.3-.4; AOR 4.5 CI 2.6-7.8 respectively). Similarly, FSWs who reported of having “collective ownership” were 2 times more likely to participate in public spaces, consistently in all three states of AP, TN, and MH (AOR 2.2 CI 1.0-4.7; AOR 2.3 CI 1.5-3.5; AOR 2.4 CI 1.1-5.3 respectively).

In the state of AP and TN, those FSWs who were participating in collectives were more likely to participate in public spaces (AOR 2.9 CI 1.7-5.0 & AOR 5.1 CI 2.6-10.3; respectively). FSWs, who had experienced “police vulnerability”, were more likely to participate in public spaces in all states of AP TN and MH, (AOR 3.6 CI 2.6-5.1; AOR 2.4 CI 0.3-2.3; AOR 3.3 CI 2.1-5 respectively).

## Discussion

This is the first of its kind study which quantitatively assessed and distinguished the theoretically defined ‘participations’ in the contexts of community mobilization of FSWs in India [6, 7]. Based on the theoretical insights, two distinct variables of FSWs’ participations were developed as participations in ‘collective spaces’ and ‘public spaces’ respectively. Data was utilized from a large scale, socio- culturally diverse and well represented high risk population of FSW which was undergoing a wider ‘community mobilization’ process under AVAHAN, India AIDS initiative. AVAHAN strategized community mobilization as a process which enables FSW communities to participate in delivering the long-term goal of reduced HIV prevalence among themselves and in the wider population, through service provision and structural interventions to reduce vulnerability [2]. The community mobilization goes beyond the HIV reduction goal with FSWs actively participating to address relevant public policy and claim their rights and entitlements further. Thus this study used an appropriate FSW population, which provided the opportunity to quantitatively assess the transforming nature of their participation from a ‘utilitarian tool’ to an ‘empowerment tool’ in the process of their community mobilization. The study has also assessed the stages of community mobilization of FSWs which influenced such transformation of their participations.

The cross sectional design of the survey do impose restrictions on capturing the dynamic and lengthy process like community mobilization interventions which is a limitation in this study. On the other hand this design has led to rapid analysis of a time consuming process, emerging with valid hypotheses. These need to be studied in practical settings of community mobilization process for FSWs and other vulnerable groups. Limited variables were used to completely represent all crucial stages of community mobilization (e.g. collective efficacy) which was a limitation.

Descriptive statistic shows that FSWs in AP and TN states had higher level of collective participations and moderate level of public participation. Comparatively MH state had very lower public participation and collective participation among FSWs. Also FSWs in MH state had shown much low level of collective identity, community identity, collective ownership and agency. Comparatively FSWs in AP and TN had higher level of all mobilization indicators. The reason behind this vast difference between MH and other two states in the mobilization and participation levels needs to be contextualized with the nature of commercial sex industry of these states. According to the IBBA data used for this study, MH was having almost equal proportion of brothel and street based FSWs in maximum districts of interventions while AP had a lower range of 1-35% of brothel based sex except in one district of with 49% brothel based FSWs. The street based sex workers in AP were in the range of 70-98% except in one district with 51% of street based FSWs. TN state had less than 10% of brothel based FSWs while remaining all were street based FSWs [18].

Eluding to the fact that MH has large brothel based sex industry and the programme data showing maximum coverage of mobilization led interventions, our findings are in contrary to the past findings which highlight that brothel settings highly favor peer cohesion and mobilization among FSWs than street sex work settings Samuels et al. [19]. We found a high level of public participation and mobilization among FSWs of AP and TN where street based sex industry thrives more. This could be explained by multiple structural and programme factors which affect the successful implementation of community mobilization interventions. Structurally it could be the differences in the conducive nature of social and political environments of the intervention area, which includes the support of local government, bureaucracy, community and other stakeholders. The differences in the nature and capacity of NGOs which implement them also act as a proximal factor affecting the implementation of mobilization. This is highlighted in the case of AP where the coastal areas with flourishing industry and agriculture have lead to the surge of street and home based sex workers. Inspite of this unfavorable environment among the less cohesive street based sex workers, the overall achievement in terms of community mobilization was impressive in AP state as reported in this study and others [20]. The reason could be explored in the developmental track record of AP state which for decades has facilitated millions of women to join self-help groups through community mobilization interventions [21, 22]. Similarly the relatively high level of mobilization and participation in TN could be explained with the fact, that this state was also a pioneer in community mobilization based developmental programmes in India [23]. Such a conducive environment in AP and TN at various levels of government, private and community could naturally have facilitated the mobilization interventions for FSWs than MH inspite of it being hosts to maximum brothel sex workers.

The reason why mobilization and participation was not much higher in MH could also be traced back to programmatic drawbacks. Past finding from the failed community mobilization program in Chennai explains the lack of knowledge and innovations about community based organizations [24]. Unlike Chennai, the successful Sonagachi project had been attributed for its programme innovations [like “mechanisms for resolving disputes with brothel madams, problems with the police and dealing with violent clients”] for its impressive success [25]. Lessons from failed mobilization interventions in South Africa also shows that the prevailing “disorganization, semi-, lawlessness, and lack of supportive structures” in the sex work environment leads to failure of mobilization interventions [26]. All these factors should be considered while assessing the status of community mobilization and participation of FSWs in different states in this study. Such arguments appear relevant in the context of MH state in this study where a high percentage of police arrest was reported by 46.5% of FSWs indicating a non- conducive and disputable sex work environments for interventions. Comparatively low level of FSWs in AP (30.5) and TN (6.6%) reported police arrests indicating a more favorable sex work environment for interventions.

Bivariate analysis shows that in MH, ‘typology of sex work’ had an inverse role in influencing FSWs’ participation based on their contexts. In MH brothel based FSWs had significantly more collective participation when compared to street based FSWs. This could be explained by the fact that the gate keepers like “Madams” could have encouraged and facilitated the ‘collective participation’ inside the brothels since this would help in keeping FSWs healthy from STIs while they are within their controls. Alternatively AP showed street based FSWs had significantly higher “collective participation” when compared to brothel based FSWs. This again reinstates the fact that although 2/3 rd of FSWs in AP state are street based - who lack the space and necessary environment for any peer training or counseling to happen in the street set ups, - the favorable structural factors of AP state which has an impressive environment for community mobilization must have favored more “collective participation”.

With regard to public participation, in MH, the brothel based FSWs showed significantly lower ‘public participation’ i.e. less than 1/10th of all. Public participation of street based FSW in MH too was significantly lower when compared to other states, but then it was higher than the brothel based FSWs in MH. This again could be understood in the context of brothel settings where the control is more in the hands of ‘madams’ or ‘managers’, who have the power and can pose sanctions on the FSWs regarding participation in any activity [25]. Thus, to safeguard their own interest and power, the brothel keepers might not be allowing participation in public spaces where the possibility of an FSW becoming more powerful exists. However, success of community mobilization interventions would lie in bringing FSWs in public spaces which could lead to prostitution policy reforms. The reforms like decriminalization could bring the confidence in FSW to refuse any client as observed after implementation of the Prostitution Reform Act 2003 of New Zealand [27]. Along with FSWs, sensitization of brothel owners is required. This sensitization need of brothel owners is further strengthened by the fact that AP state having more of street based sex work industry had significantly higher level of “public participation” possibly due to absence of any constraints from powerful ‘madams’ or brothel owners. Another interesting observation was regarding brothel based FSWs in AP who showed significantly higher “public participation” as compared to MH state, even though it was lesser when compared to street based sex workers in AP. As indicated before the reason could be attributed to the track record of AP state which pioneered in the participatory women self help group movement in India and a strong program back ground conducive for mobilizations interventions across settings.

While the bivariate association of factors like duration of sex work, literacy and age with increased public/collective participation of FSWs are self explanatory, other factors like occupation, and debt status of FSWs which exhibited such bivariate relationship needs to be assessed specifically further. Overall in this study FSWs characteristics, community mobilization and vulnerability status had shown varied levels of bivariate association with their collective and public participations, which strengthens their distinct nature as hypothesized by this study.

Our regression analysis provides insights, in understanding the participation of FSWs as a dynamic process which inherently evolves together with the community mobilization process.

### Collective and community identity of FSWs and their relation with their collective and public participation

While the collective identity i.e. the initial collective stage was found to have no significant influence on FSW’s public participation in any of the states, yet, community identity, (the later community engagement stage of mobilization process) was found influencing public participation in two states except in MH (where generally all the levels of mobilization was less attained). Strikingly opposite to this was the finding that FSW’s participation in ‘collective spaces’ was influenced by ‘collective identity’ consistently in all the three states. This revealed the contextual difference of the FSW’s identity and participation which is a new finding from this study. The collective identity attained by the membership in an informal group (Self Help Group), which has a limited mission of disease prevention, logically has no role in influencing the ‘public participation’. Rather the community identity which was gained by being a member of more organized and centralized organizations beyond the collective spaces was rather influencing ‘public participation’.

### Collective agency of FSWs and its relation with their collective and public participation

Another insight gained was that, while ‘collective agency’ was found to be 2–4 times more likely to be associated with FSWs’ public participation, it showed no association with collective participation in any of the states. The ‘collective agency’ is a known indicator of community mobilization in terms of FSW’s bargaining with potential stakeholders for their rights and this indicator has been widely used in the context of safe sex practices [5]. However, our study reveals that ‘collective agency’ would be a more suitable indicator in the context of sex workers empowerment instead of safe sex practices.

### Collective ownership of FSWs and its relation with their collective and public participation

Unlike the collective-community identities and collective agency, the collective ownership status of the FSWs (being peer educators/ volunteers) was found to influence both public and collective participation by them. Peer education has been the corner stone of community mobilization led HIV intervention programmes, and peer educators remain the most active participants in that mobilization process exhibiting qualities of ownership and leadership [28]. This study reaffirms the unique and principle role of peer educators in the community mobilization process in all the states, in which FSWs as peer educators actively participate in both collective and public spaces, thus setting themselves in a leading participant role for other FSWs to follow.

### Police vulnerability of FSWs and its relation with collective and public participation

Apart from the factors related to community mobilization, experience of police vulnerability was having a consistent influence on FSW’s public participation in all states, which explains the underlying need for social capital, protection and power among vulnerable sex workers. In this study, police vulnerability acted as a ‘push’ factor for FSWs to move towards public space to seek power and rights against structural barriers. This finding matches with other studies where FSWs were able to successfully regulate powerful state actors like police, with their powers gained through community mobilization [29, 30]. Results also show that collective participation of FSWs influences their public participation and vice versa, since the former one (collective participation) naturally develops as the latter one (public participation) and thus have mutual influences.

The framework of this study considered all the successive stages of community mobilizations to be none overlapping and separate stages, which is not possible in real life situation. However, such limitations were overlooked to theoretically establish the contextual differences of the community mobilization process and participation. Participation in ‘collective’ and public spaces’ can serve as new indicators, and will help assess the community mobilization process which largely remains much abstract, and difficult to measure precisely [31]. Both the indicators of participation symbolize the passage of community members from their individual issues of disease and safety towards broader societal issues and engagement with structural barriers [32, 33]. Specifically the new indicator ‘FSW’s participation in public spaces’ which is also noted as ‘public visibility of sex workers’ [34], quantifies a critical step in the community mobilization process, which has the potential to further evolve into complete social empowerment of FSWs.

## Conclusion

This study has assessed the specific nature of ‘participations’ of FSWs and revealed its relation with the specific ‘contexts’ and stages of community mobilization, which would lead to better understand the dynamics of community mobilization as a process. In particular “public participation” of FSW could indicate the level of their empowerment strivings against structural barriers through which they negotiate with the stigmatizing social norms and gain their social capital. The participation indicators could help advance the evidence base to understand the participatory mobilization led intervention programmes at community and societal levels. The study emphasizes the need to further specify and test these sensitive indicators, to validly measure the complex process of participatory mobilization. These indicators could be used for effectively monitoring the community mobilization programmes among HRGs in the intervention settings.
